# Claudin-2 Knockout by TALEN-Mediated Gene Targeting in MDCK Cells: Claudin-2 Independently Determines the Leaky Property of Tight Junctions in MDCK Cells

**DOI:** 10.1371/journal.pone.0119869

**Published:** 2015-03-17

**Authors:** Shinsaku Tokuda, Mikio Furuse

**Affiliations:** 1 Division of Cell Biology, Department of Physiology and Cell Biology, Kobe University Graduate School of Medicine, Kobe 650–0017, Japan; 2 Division of Cerebral Structure, National Institute for Physiological Sciences, Okazaki 444–8787, Japan; 3 Department of Physiological Sciences, The Graduate University for Advanced Studies (SOKENDAI), Okazaki 444–8585, Japan; University of Chicago, UNITED STATES

## Abstract

Tight junctions (TJs) regulate the movements of substances through the paracellular pathway, and claudins are major determinants of TJ permeability. Claudin-2 forms high conductive cation pores in TJs. The suppression of claudin-2 expression by RNA interference in Madin-Darby canine kidney (MDCK) II cells (a low-resistance strain of MDCK cells) was shown to induce a three-fold increase in transepithelial electrical resistance (TER), which, however, was still lower than in high-resistance strains of MDCK cells. Because RNA interference-mediated knockdown is not complete and only reduces gene function, we considered the possibility that the remaining claudin-2 expression in the knockdown study caused the lower TER in claudin-2 knockdown cells. Therefore, we investigated the effects of claudin-2 knockout in MDCK II cells by establishing claudin-2 knockout clones using transcription activator-like effector nucleases (TALENs), a recently developed genome editing method for gene knockout. Surprisingly, claudin-2 knockout increased TER by more than 50-fold in MDCK II cells, and TER values in these cells (3000–4000 Ω·cm^2^) were comparable to those in the high-resistance strains of MDCK cells. Claudin-2 re-expression restored the TER of claudin-2 knockout cells dependent upon claudin-2 protein levels. In addition, we investigated the localization of claudin-1, -2, -3, -4, and -7 at TJs between control MDCK cells and their respective knockout cells using their TALENs. Claudin-2 and -7 were less efficiently localized at TJs between control and their knockout cells. Our results indicate that claudin-2 independently determines the ‘leaky’ property of TJs in MDCK II cells and suggest the importance of knockout analysis in cultured cells.

## Introduction

In multicellular organisms, epithelia act as a barrier to the external environment. Epithelial cells adhere to each other through complexes that form junctions between the cells, and the tight junction (TJ) is located in the most apical part of the complexes [1]. TJs regulate the movement of substances through paracellular pathways of the various permeabilities found among epithelia (barrier function), contributing to the generation and maintenance of the proper internal environment required for organ function [2,3]. The major determinants of permeability in TJs are claudins, a large family (27 members in mammals) of integral membrane proteins identified in 1998 [4–6]. Epithelia express multiple different claudins and the expression pattern of claudins is thought to be responsible for the variety of different permeabilities in TJs [7,8].

Of the claudins, claudin-2 barrier properties in cultured epithelial cells have been most well studied. The exogenous expression of claudin-2 in Madin-Darby canine kidney (MDCK) I cells, a high-resistance strain of MDCK cells that lack claudin-2 expression, decreased transepithelial electrical resistance (TER), a reciprocal of the ion conductance across the epithelia, by more than 10-fold, and transformed so-called ‘tight’ epithelia into ‘leaky’ epithelia [9,10]. Amasheh et al. and afterward other groups demonstrated that claudin-2 forms high conductive pores with cation selectivity in TJs [10–12]. In contrast, the suppression of claudin-2 expression by RNA interference (knockdown) in MDCK II cells, a low-resistance strain of MDCK cells that express endogenous claudin-2, induced a three-fold increase in TER [13,14]. However, the values of TER in claudin-2 knockdown MDCK II cells were markedly lower (130–250 Ω·cm^2^) than those in high-resistance strains of MDCK cells (> 1000 Ω·cm^2^). One explanation for the low values of TER in claudin-2 knockdown MDCK II cells is the differential expression of claudins other than claudin-2, between claudin-2 knockdown MDCK II cells and high-resistance MDCK strains. However, because RNA interference-mediated knockdown is not complete and only reduces gene function, another possibility is that claudin-2 pores formed from residual claudin-2 expression during knockdown could still have significant effects on TER, resulting in lower TER in claudin-2 knockdown cells.

The complete elimination of gene function through changes in the genetic code (knockout) is an ideal method for the analysis of genes. Recently, genetic engineering has been improved by the use of zing-finger nucleases (ZFNs), transcription activator-like effector nucleases (TALENs), and the clustered regularly interspaced short palindromic repeats (CRISPR)/Cas system [15–17]. These techniques are being used increasingly to knockout genes in model organisms and cultured cells [18], but to date there have been few reports comparing the knockout analysis of target genes using these methods with knockdown analysis in cultured cells [19]. Because TALENs are easy to construct compared with ZFNs [20], and the CRISPR/Cas system may have a problem with specificity [18], we chose TALENs to knockout claudin-2 in MDCK II cells to investigate its functions in detail.

Transcription activator-like effectors (TALEs) are natural bacterial proteins secreted by *Xanthomonas* sp., which contain tandem repeats of DNA-binding domains that recognize specific nucleotides [21]. TALENs are artificial nucleases generated by fusing a *Fok*I DNA cleavage domain to TALEs. Two TALENs that recognize the left and right arms of the target site form a functional *Fok*I dimer and induce DNA double-strand breaks at the target site. Normally, DNA double-strand breaks are repaired by non-homologous end joining pathways, resulting in the introduction of nucleotide mismatches, insertions, or deletions, and functional gene knockout [16].

In this study, we established claudin-2 knockout clones in MDCK II cells using TALENs in a similar manner as described previously [19]. Surprisingly, claudin-2 knockout increased TER by more than 50-fold in MDCK II cells. Claudin-2 expression restored the TER of claudin-2 knockout cells dependent upon claudin-2 protein levels. Our results indicate that claudin-2 independently determines the ‘leaky’ property of TJs in MDCK II cells and suggest the importance of knockout analysis in cultured cells.

## Materials and Methods

### Cells, antibodies and reagents

MDCK II cells were provided by Dr. Masayuki Murata [9]. MDCK I cells were provided by the late Dr. Shoichiro Tsukita (Kyoto University) and maintained in our laboratory [9]. Cells were grown in DMEM (high glucose) supplemented with 5% fetal bovine serum.

Mouse anti-ZO-1 monoclonal antibody (mAb) (T8/754), rat anti-occludin mAb (MOC37), rabbit anti-claudin-2 polyclonal antibody (pAb), and rabbit anti-claudin-4 pAb were characterized as described previously [5,22–24]. Rabbit anti-ZO-2 pAb (38–9100), rabbit anti-ZO-3 pAb (36–4100), rabbit anti-claudin-1 pAb (51–9000), mouse anti-claudin-2 mAb (32–5600), rabbit anti-claudin-3 pAb (34–1700), mouse anti-claudin-4 mAb (32–9400), rabbit anti-claudin-7 pAb (34–9100), and alexa fluor 488 phalloidin (A12379) were purchased from Invitrogen. Rabbit anti-FLAG pAb (PM020) and rat anti-GFP mAb (D153–3) were purchased from Medical and Biological Laboratories. Mouse anti-FLAG mAb (018–22381) was purchased from Wako. Rabbit anti-nonmuscle myosin heavy chain II-B (MHC-B) pAb (PRB-445P) was purchased from Covance. Mouse anti-E-cadherin mAb (ECCD-2; M108) was purchased from Clontech. Fluorescein isothiocyanate-dextran (FITC-dextran) was purchased from Sigma-Aldrich. Fluorescein (16106–82) was purchased from Nacalai tesque.

### Construction of TALENs and establishment of knockout clones

TALENs were constructed following the detailed instruction provided by the TALE Toolbox kit from the Zhang laboratory [20] (Addgene, #1000000019). To establish claudin-2 knockout clones, a pair of TALEN constructs for the claudin-2 knockout were cloned into a mammalian expression vector pCAGGS [25] with a neomycin resistance gene and puromycin resistance gene, respectively. Cells were seeded in a 6-well plate (Falcon) at a density of 4 × 10^4^ cells/well and these vectors were transfected into cells 2 h after seeding using Lipofectamine LTX with Plus Reagent (Invitrogen) following the manufacturer’s protocol. Then 500 μg/ml G418 and 5 μg/ml puromycin were administered for 4 h on the day following the transfection. Remaining clones were isolated and screened for claudin-2 depletion by immunocytochemistry.

### cDNA cloning and plasmid construction

cDNA encoding dog claudin-2 described previously [9] was cloned into pCAGGS with N-terminal 1×FLAG (DYKDDDDK) tag and 2×Strep II (WSHPQFEK) tags and pCAGGS without tag. To establish stably expressing clones, the vectors were transfected into cells and stable clones were selected in standard media supplemented with 500 μg/ml G418.

### DNA sequencing analysis

DNA sequencing was performed using the dideoxy chain termination method with BigDye Terminator version 3.1 Cycle Sequencing Kit (Applied Biosystems) and results were analyzed by the Applied Biosystems 3130 Genetic Analyzers (Applied Biosystems). The chromatograms of the sequence results were analyzed using Peak Scanner Software 2 (Applied Biosystems).

### PCR amplification of genomic DNA

Genomic DNA were isolated by the Hot-shot method [26] and subjected to PCR for the amplification of TALEN targeting site in claudin-2 gene (Forward: 5′-ACCCACAGACACTTGTAAGG-3′; Reverse: 5′-CCAACGAAGAGATCGCACTG-3′) and TALEN C-terminal region (Forward: 5′-CTGCGGCACAAATTGAAATA-3′; Reverse: 5′-ATGAGCGGAAATTGATCTCG-3′). The PCR products of TALEN targeting site in claudin-2 gene were directly subjected to sequence analysis. For claudin-2 knockout clones 2–5, PCR products were cloned into pCAGGS and subjected to sequence analysis.

### Immunocytochemistry

Immunocytochemistry was performed on cells cultured on 12-mm-diameter Transwell filter inserts with a 0.4-μm pore size (Corning, Corning, NY). Cells were plated at a density of 2 × 10^5^ cells/cm^2^ and cultured for 4 d unless otherwise noted. Filter inserts were fixed in 1% paraformaldehyde for 10 min at room temperature or in 100% methanol for 10 min at −20°C. Then filters were permeabilized in a solution of 0.2% (w/v) Triton X-100 (EMD Biosciences) in PBS for 60 min, blocked with 2% bovine serum albumin, and incubated with a primary Ab followed by a fluorescence-labeled secondary Ab. Filamentous actin (F-actin) was visualized using alexa fluor 488 phalloidin (Molecular Probes, Invitrogen, A12379). Samples were imaged on a Zeiss LSM700 confocal microscope using a 63× Plan Apo lens. Contrast adjustment was generated using Adobe Photoshop (ver. 7.0).

### Immunoblotting

For immunoblotting of cell lysates, cells cultured on Transwell filter inserts were scraped into Laemmli SDS sample buffer and boiled for 5 min. The proteins were separated by one-dimensional SDS-PAGE and electrotransferred from the gels to PVDF membranes followed by the incubation with primary Abs. The bound Abs were detected using HRP-linked secondary Abs and visualized by enhanced chemiluminescence (ECL Prime Kit; GE Healthcare). The signal intensity of claudin-2 bands were quantified using ImageJ 1.43u (available at http://rsb.info.nih.gov/ij; developed by Wayne Rasband, National Institutes of Health, Bethesda, MD).

### Barrier assays: electrophysiological measurements and tracer flux

Electrophysiological studies were performed as described previously [19]. Cells were plated at a density of 2 × 10^5^ cells/cm^2^ on Transwell filter inserts, and electrical resistance across the cell monolayer was measured using Millicell-ERS epithelial volt-ohm meter (Millipore) every day for 6 d, and transepithelial electrical resistance (TER) was determined by the subtraction of the resistance of the blank filter. To determine the ion permeability of Na^+^ (*P*_Na_) and Cl^−^ (*P*_Cl_) across the epithelia, dilution potentials and TER of cell monolayers cultured for 6 d were measured with solution A [140 mM NaCl, 5 mM glucose, 5 mM KCl, 1 mM MgCl_2_, 1 mM CaCl_2_ and 10 mM HEPES-NaOH (Ph 7.4)] in the apical side and solution B [70 mM NaCl, 130 mM sucrose, 5 mM glucose, 5 mM KCl, 1 mM MgCl_2_, 1 mM CaCl_2_ and 10 mM HEPES-NaOH (pH 7.4)] in the basolateral side at 37°C, and electrical potentials and resistance of the blank filter under the same condition was subtracted. Stability of electrical potentials and resistance was confirmed by repetitive measurements for at least 5 minutes. The *P*_Na_/*P*_Cl_ ratio was calculated using the Goldman–Hodgkin–Katz equation. The values of *P*_Na_ and *P*_Cl_ were then calculated from the TER and *P*_Na_/*P*_Cl_ using the Kimizuka–Koketsu equation [27].

For measurements of tracer flux, cell monolayers cultured for 6 d were incubated in solution A with 0.2 mM fluorescein or FITC-dextran in the basolateral side for 1 h, and the solutions in the apical side were collected. Fluorescence of the solutions at 518 nm was measured using a fluorescence spectrophotometer (F-4500; Hitachi High-Tech) with an excitation wavelength of 488 nm, and amounts of FITC–dextran were determined by extrapolation from a standard curve of known fluorescein or FITC–dextran concentrations using linear regression. The permeability of fluorescein and FITC-dextran was defined as (dQ/dt)/AC_0_ [19].

### Quantification of the localization of TJ proteins at the TJs

We quantified the signal intensity of TJ proteins at TJs using Image J 1.43u in a similar manner as previously described [28]. We performed double staining for the target proteins with TJ marker proteins (ZO-1, ZO-3 or occludin), and single confocal images of double-stained monolayers at the level of TJs were captured. Images were opened in Image J 1.43u, and immunofluorescence signals of TJ marker proteins were traced with 0.5 μm-wide freehand lines to build the region of interest (ROI; S1 Fig.). We traced five sides of control cells, knockout cells and the boundary between control and knockout cells in one image, and integrated density of pixel gray values of ROI was calculated (D_CTL_, D_KO_ and D_Boundary_). To estimate the localization of claudins other than claudin-2 at TJs in claudin-2 knockout cells, the relative intensity of each claudin was calculated as D_KO_/D_CTL_. To investigate the localization of claudin-2 at TJs between control and claudin-2 knockout cells, D_KO_ was subtracted from D_Boundary_ and D_CTL_ to eliminate the effects of non-specific signals, and the relative intensity was calculated as (D_Boundary_—D_KO_)/(D_CTL_—D_KO_). We also conducted the knockouts of TJ proteins other than claudin-2 by TALENs, and investigated the localization of them at the TJs between control and their knockout cells in the same manner.

### Statistical analysis

Data are represented as means ± standard error of the mean. We performed Bartlett’s test to test variance, and when the variance was confirmed not to be unequal, we performed Bonferroni correction for multiple comparisons. P < 0.05 was considered statistically significant.

## Results

### Construction of TALENs for claudin-2 gene knockout in MDCK II cells

**Fig 1:**
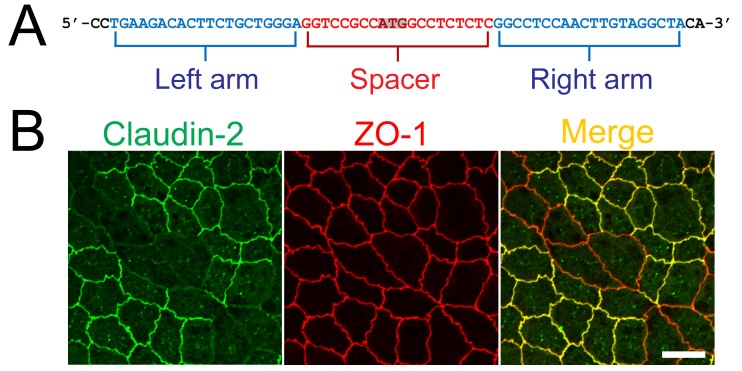
Construction of TALENs and claudin-2 gene knockout in MDCK II cells. (A) TALEN binding sites in the claudin-2 gene. The left and right arms of TALEN targeting site are indicated in blue and the spacer region is indicated in red. The initiating codon within the spacer region is shaded. (B) Immunofluorescence analysis of claudin-2 and ZO-1 in MDCK II cells transfected with TALEN constructs for claudin-2 gene knockout. After transfection, cells were subcultured on filter inserts for 4 days before analysis. Claudin-2 staining was completely lost at cell-cell contacts in claudin-2 knockout cells. Scale bar = 10 μm.

To generate TALEN DNA constructs to knockout the claudin-2 gene in MDCK II cells, we designed TALENs targeting the left and right arms of the initiating codon in the canine claudin-2 gene (Fig. 1A), and constructed TALENs as previously described [20]. The TALEN constructs were transfected into MDCK II cells, and immunofluorescence analysis of claudin-2 revealed complete loss of claudin-2 staining at cell-cell contacts in some regions, indicating the validity of the TALEN constructs for claudin-2 gene knockout in MDCK II cells (Fig. 1B).

### Establishment of claudin-2 knockout clones in MDCK II cells

Next, we established claudin-2 knockout clones in MDCK II cells. To increase the efficiency of the selection of knockout clones, we cloned a pair of TALEN DNA constructs for claudin-2 knockout into mammalian expression vectors with neomycin- and puromycin-resistance genes. These vectors were transfected into MDCK II cells, and G418 and puromycin were transiently administered as described previously [19]. The remaining cell colonies were screened by immunofluorescence microscopy, followed by limiting dilution culture, and we established five independent clones.

**Fig 2:**
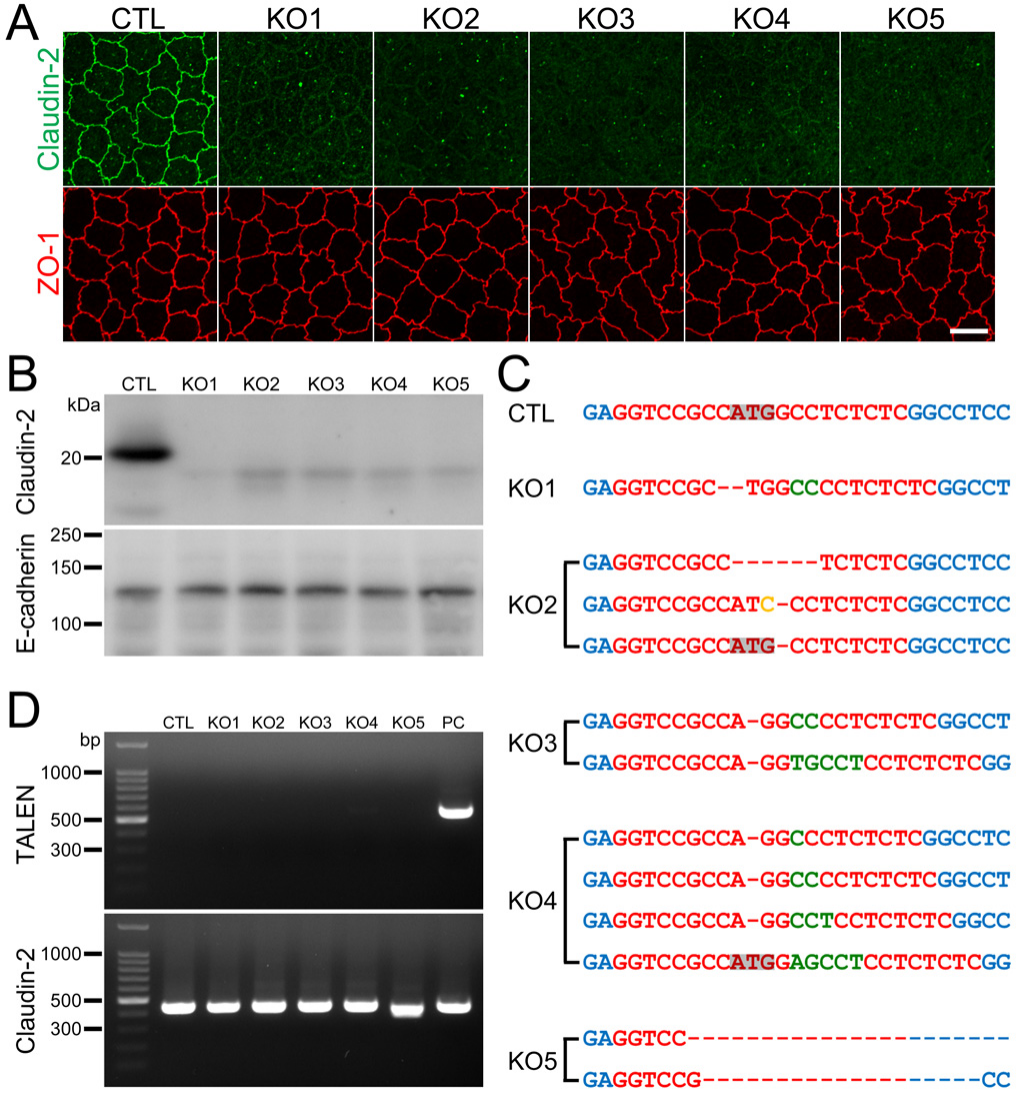
Establishment of claudin-2 knockout clones in MDCK II cells. (A) Immunofluorescence analysis of claudin-2 and ZO-1 in control MDCK II cells (CTL) and claudin-2 knockout clones (KO 1–5). Claudin-2 staining at cell-cell contacts was completely lost in claudin-2 knockout clones. Scale bar = 10 μm. (B) Immunoblots of claudin-2 and E-cadherin in control MDCK II cells and claudin-2 knockout clones. A claudin-2 band of ~22 kDa was absent in claudin-2 knockout clones, but faint bands of a lower molecular weight than wild-type claudin-2 band were observed. (C) DNA sequences of the TALEN targeting site in each allele of claudin-2 knockout clones. One type of mutation was found in the alleles of claudin-2 knockout clone 1 (KO 1), two types in the alleles of clones 3 and 5 (KO 3 and 5), three types in the alleles of clone 2 (KO 2), and four types in the alleles of clone 4. Dashes indicate loss of nucleotides, green letters indicate additional nucleotides, and a yellow letter indicates an altered nucleotide. Loss of initiating codon or frame shift was confirmed for all alleles. (D) Genomic PCR analysis of control and claudin-2 knockout clones using primers for TALENs and claudin-2 DNAs. A clone stably expressing TALEN was used as a positive control (PC). None of the PCR products for TALENs was detected in claudin-2 knockout clones.

Immunofluorescence analysis revealed the complete loss of claudin-2 staining at cell-cell contacts in all clones (Fig. 2A). Immunoblot analysis showed the disappearance of a ~22 kDa band representing claudin-2 in these clones, but faint bands appeared at a molecular weight lower than that of wild-type claudin-2 (Fig. 2B). The faint bands were detected by two different antibodies that recognize the claudin-2 C-terminal region, indicating the specificity of these bands for this region (S2 Fig.). Since the open reading frame of canine claudin-2 gene has an in-frame ATG sequence at the 24th codon, we thought that the faint bands might reflect an artificial peptide produced by translation using the 24th ATG codon of claudin-2 gene as its initiating codon. To confirm the mutations of the TALEN targeting site in the claudin-2 gene, PCR products from this site in the clones were directly subjected to DNA sequencing analysis. Chromatograms of the sequences showed a single peak array in clone 1 and mixed peak arrays in clones 2–5 (S3A Fig.). We therefore cloned the PCR products of clones 2–5 into a plasmid vector for sequence analysis. Two patterns were observed in the chromatograms of the sequences for clones 3 and 5, three patterns for clone 2, and four patterns for clone 4. A comparison of their peak arrays revealed that their mixed peak arrays comprised these chromatograms, respectively (S3A Fig.). Sequence analysis for each allele revealed a loss of the initiating codon or a frame shift in all alleles (Fig. 2C and S3B Fig.). These results indicated successful claudin-2 gene knockout in these clones.

We also confirmed whether the TALEN constructs transfected into the clones were integrated into the chromosome. We performed genomic PCR using primers for the TALEN C-terminal region. A clone stably expressing TALEN established in a previous study was used as a positive control [19]. No PCR products from the clones had detectable bands of 558 bp representing the TALENs (Fig. 2D), suggesting the TALEN constructs were not integrated into the chromosome in these clones.

### Effect of claudin-2 knockout on the localization of other claudins

**Fig 3:**
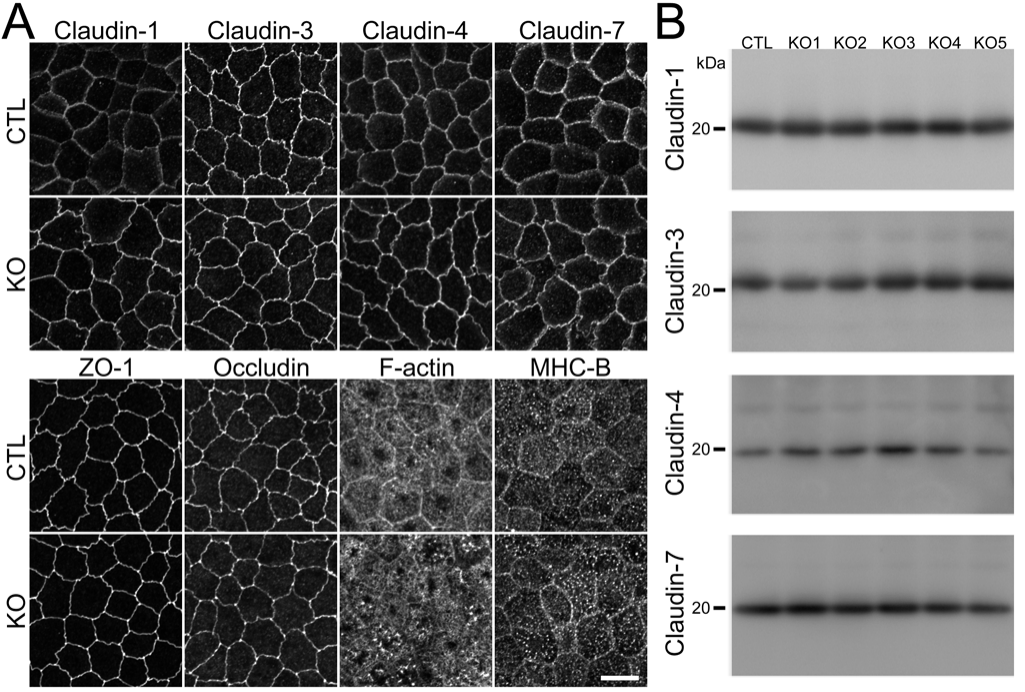
Effects of claudin-2 knockout on the localization of other TJ proteins and cytoskeleton. (A) Immunofluorescence analysis of claudin-1, -3, -4, -7, ZO-1, occludin, F-actin, and myosin heavy chain II-B (MHC-B) in control and claudin-2 knockout cells. Claudin-1 and -7 showed a tendency to be more clearly localized at TJs in claudin-2 knockout cells. Scale bar = 10 μm. (B) Immunoblots of claudin-1, -3, -4, and -7 in control MDCK II cells and claudin-2 knockout clones. Similar expression levels of claudin-1, -3, -4, and -7 were observed in control cells and knockout clones.

**Fig 4:**
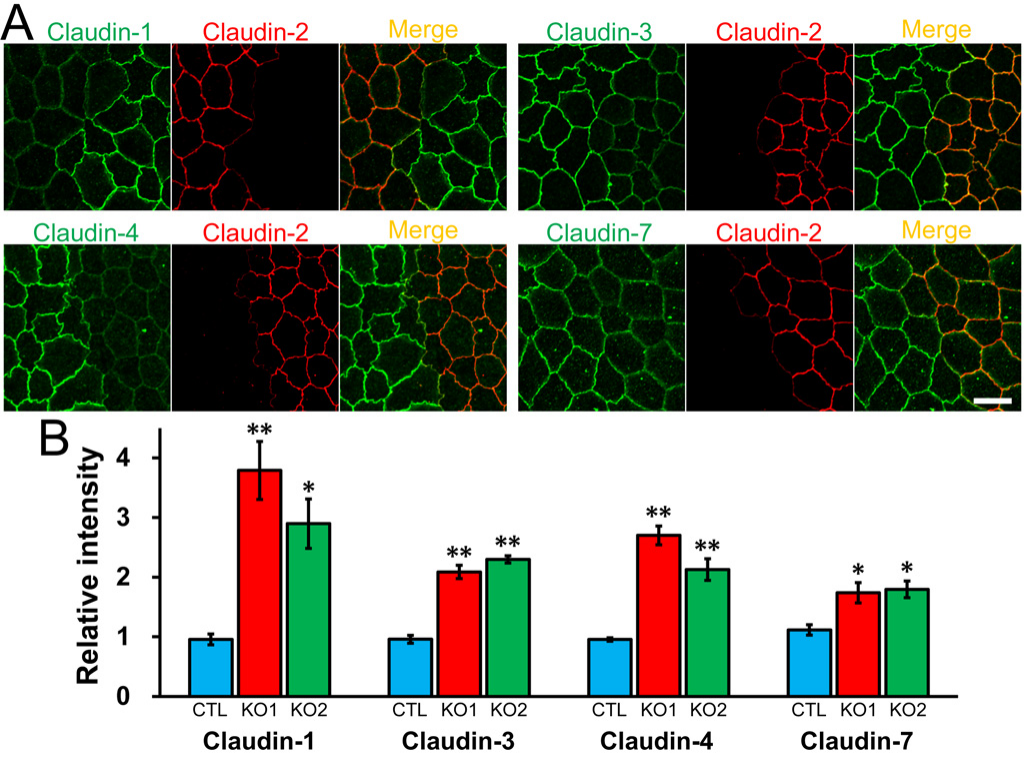
Effects of claudin-2 knockout on the localization of other claudins. (A) Immunofluorescence analysis of claudins in co-culture of control MDCK II cells and claudin-2 knockout clone 1 (KO 1). Claudin-1, -3, -4, and -7 showed clearer and stronger signals at TJs in claudin-2 knockout cells than in control cells. Scale bar = 10 μm. (B) Quantification analysis of signal intensity of claudins at TJs in control MDCK II cells and claudin-2 knockout clones. The signal intensity of claudins at TJs in control cells and claudin-2 knockout clones was measured as described in *Materials and Methods*, and the relative signal intensity of each claudin was calculated as the ratio of the signal intensity in control cells (CTL) and claudin-2 knockout clones (KO 1 and 2) to the signal intensity in control cells. N = 4 for each experiment. * *p* < 0.05, ** *p* < 0.01 compared with control.

**Fig 5:**
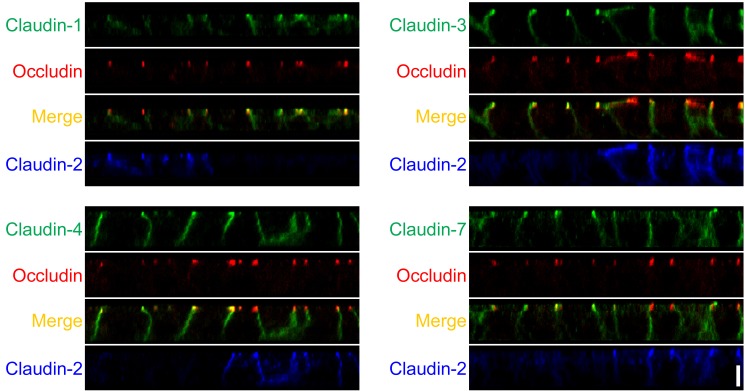
Localization of claudins in z-axis plane in control and claudin-2 knockout cells. Immunofluorescence analysis of claudins and occludin in co-culture of control MDCK II cells and claudin-2 knockout clone 1 (KO 1) in z-axis plane. The signals of claudin-1, -3, -4 and -7 were stronger at TJs in claudin-2 knockout cells compared with control cells, and lateral localization of these claudins was similar between control and claudin-2 knockout cells. Scale bar = 5 μm.

Claudins are the major constituents of TJ strands and the expression pattern of claudins is thought to determine the permeability of TJs [7,8]. Because MDCK II cells express claudin-1, -3, -4, and -7 as well as claudin-2 [13,19], we investigated the effects of claudin-2 knockout on the localization and expression levels of the other claudins. Immunofluorescence analysis revealed that claudin-1 and -7 showed a tendency to be more clearly localized in cell-cell contacts at the TJ level in claudin-2 knockout cells compared with control cells (Fig. 3A). In contrast, the localization of ZO-1 (scaffold protein in TJs), occludin (integral membrane protein in TJs), F-actin, and myosin was similar between control and claudin-2 knockout cells (Fig. 3A). To confirm the effects of claudin-2 knockout on the localization of other claudins, claudin-2 knockout clones 1 and 2 were co-cultured with control cells, and observed by immunofluorescence microscopy (Fig. 4A and S4 Fig.). Claudin-1, -3, -4, and -7 had clearer and stronger signals at cell-cell contacts at the TJ level in claudin-2 knockout clones compared with control cells. In z-axis plane, the signals of claudin-1, -3, -4 and -7 were stronger at TJs in claudin-2 knockout cells compared with control cells, and lateral localization of these claudins was similar between control and claudin-2 knockout cells (Fig. 5 and S5 Fig.). To evaluate the degree of claudin localization at TJs, the signal intensity of claudins overlapping with ZO-3 signals (scaffold protein in TJs) was quantified in control and claudin-2 knockout cells as described by Yu et al. [28]. The signal intensity of claudin-1, -3, -4, and -7 at TJs was significantly higher in claudin-2 knockout cells than in control cells (Fig. 4B). On the other hand, the protein expression levels of claudins other than claudin-2 were similar between control cells and claudin-2 knockout clones (Fig. 3B). These results indicate that claudin-2 knockout increases the localization of other claudins at TJs without a significant effect on protein expression levels.

### Effect of claudin-2 knockout on TJ barrier function

**Fig 6:**
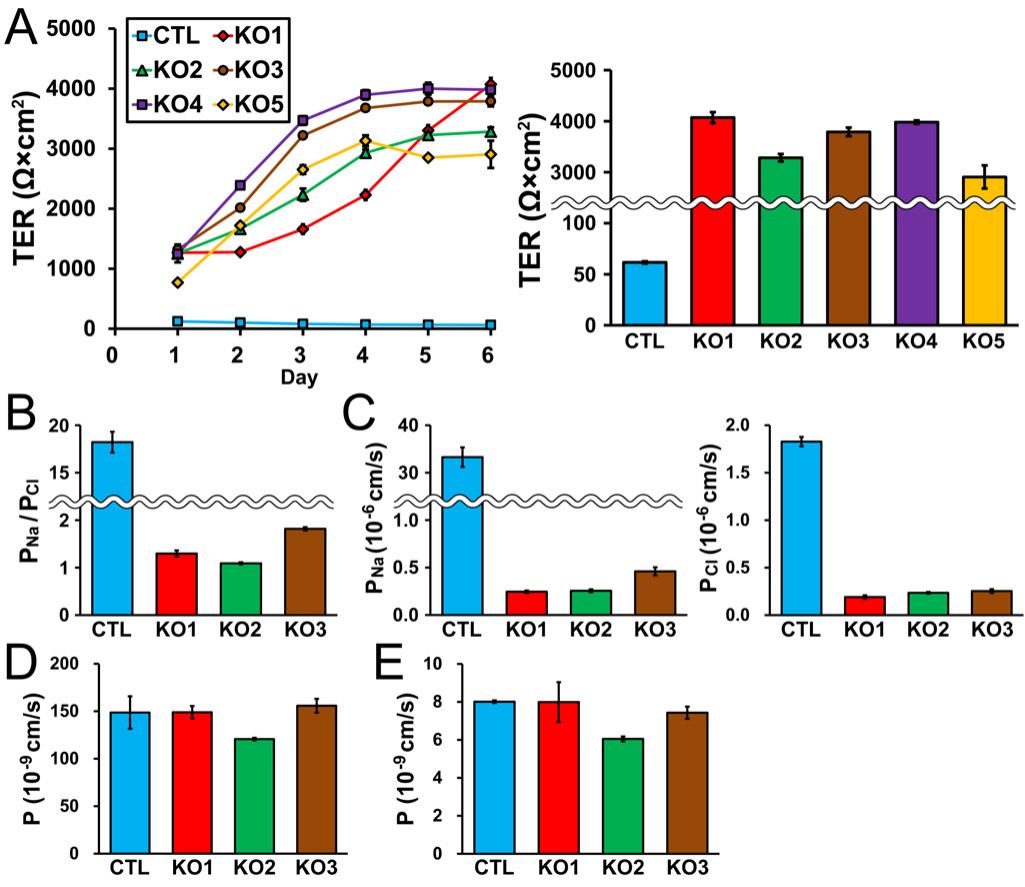
Effects of claudin-2 knockout on the barrier properties of TJs. (A) Time course of TER and TER values at 6 days after seeding on filter inserts in control cells and claudin-2 knockout clones. Claudin-2 knockout clones showed much higher TER than control cells 1 day after seeding on filter inserts and the TER further increased with time. TER values of claudin-2 knockout clones at 6 days after seeding on filter inserts were more than 50-fold higher than control cells. (B) Charge selectivity (ratio of *P*_Na_ to *P*_Cl_: *P*_Na_/*P*_Cl_) in control cells and claudin-2 knockout clones. Claudin-2 knockout clones showed much lower values of *P*_Na_/*P*_Cl_ than control cells. (C) *P*_Na_ and *P*_Cl_ in control cells and claudin-2 knockout clones. *P*_Na_ in claudin-2 knockout clones was approximately 1% of that in control cells and *P*_Cl_ in claudin-2 knockout clones was approximately 12% of that in control cells. (D and E) Flux of fluorescein (D) and 4 kDa FITC-dextran (E) in control cells and claudin-2 knockout clones. Claudin-2 knockout had no significant effects on the flux of fluorescein and 4 kDa FITC-dextran. N = 3–5 for each experiment.

Claudin-2 forms high conductive cation pores in TJs, and the suppression of claudin-2 expression by RNA interference in MDCK II cells induced a three-fold increase in TER [13,14]. We therefore measured TER in control cells and claudin-2 knockout clones. Control MDCK II cells had a TER value of 122 ± 41 Ω·cm^2^ 1 day after seeding on filter inserts, and the TER gradually decreased with time similar to previous studies [19,29] (Fig. 6A). In contrast, claudin-2 knockout clones showed much higher TER values 1 day after seeding on filter inserts (average of mean values in knockout clones, 1170 ± 101 Ω·cm^2^), and the TER further increased with time. TER values in claudin-2 knockout clones at 6 days after seeding were more than 50-fold higher than in control cells (average of mean values in knockout clones, 3606 ± 222 Ω·cm^2^ vs control cells, 61.6 ± 0.7 Ω·cm^2^; Fig. 6A). We also measured Na^+^ and Cl^-^ permeability across the epithelia (*P*_Na_ and *P*_Cl_). Control MDCK II cells showed high cation selectivity (ratio of *P*_Na_ to *P*_Cl_: *P*_Na_/*P*_Cl_) consistent with previous studies [13,19] (Fig. 6B). In contrast, claudin-2 knockout clones showed much lower values of *P*_Na_/*P*_Cl_ (average of mean values in knockout clones, 1.40 ± 0.22 vs control cells, 18.21 ± 0.49). *P*_Na_ in claudin-2 knockout clones was approximately 1% of that in control cells (average of mean values in knockout clones, 0.32 ± 0.07 × 10^–6^ cm/s vs control cells, 33.28 ± 0.92 × 10^–6^ cm/s), and *P*_Cl_ in claudin-2 knockout clones was approximately 12% of that in control cells (average of mean values in knockout clones, 0.23 ± 0.02 × 10^–6^ cm/s vs control cells, 1.83 ± 0.02 × 10^–6^ cm/s; Fig. 6C).

On the other hand, claudin-2 knockout had no significant effect on the permeability of fluorescein (332 Da), a divalent midsized anion, or 4 kDa FITC-dextran consistent with a previous study [14] (Fig. 6D and E). These results indicate that claudin-2 knockout increases TER by more than 50-fold with a decrease of cation selectivity in MDCK II cells.

### Claudin-2 re-expression restores the localization of other claudins and TJ barrier function of claudin-2 knockout cells

**Fig 7:**
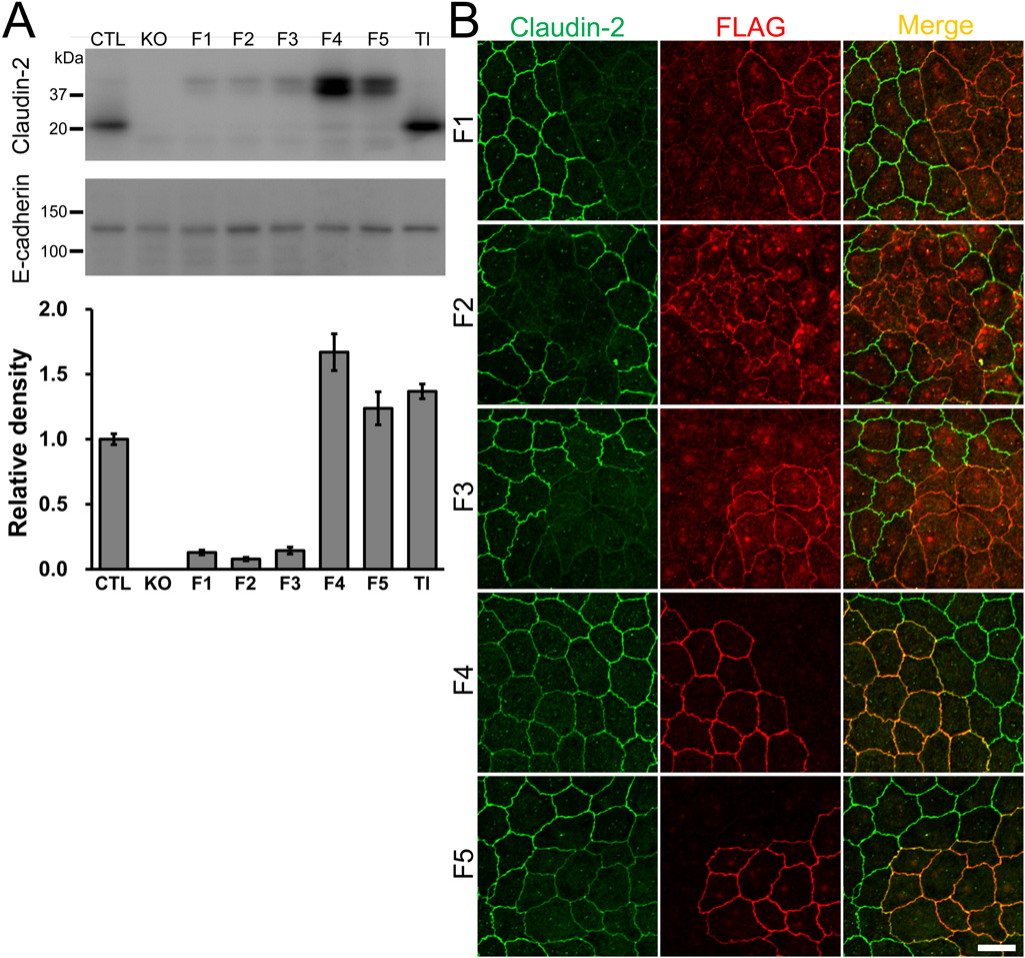
Characterization of claudin-2 knockout clones expressing exogenous claudin-2. (A) Immunoblots of claudin-2 and E-cadherin in control MDCK II cells and claudin-2 knockout clones expressing exogenous claudin-2. Claudin-2 cDNA was transfected into claudin-2 knockout clone 1 (KO 1), and clones expressing FLAG tagged claudin-2 (clones F1–5) and tagless claudin-2 (clone Tl) were established. The signal intensity of claudin-2 bands was quantified, and relative signal intensity was calculated as the ratio of the signal intensity in each clone to that in control cells. N = 4 for each experiment. (B) Immunofluorescence analysis of claudin-2 and FLAG in co-culture of control MDCK II cells and F1–5 clones. Claudin-2 staining was barely detectable at TJs in F1–3 clones, whereas claudin-2 staining in the F4 and 5 clones was clearly detected at TJs but signals were weaker compared with control cells. Scale bar = 10 μm.

Next, we performed rescue experiments. To investigate the relationship between claudin-2 protein levels and electrophysiological properties in MDCK II cells, we established clones expressing various amounts of claudin-2 using canine claudin-2 cDNA tagged with FLAG at the N-terminus (clones F1–5) and tagless claudin-2 cDNA (clone Tl). Immunoblot analysis revealed that F1–3 clones expressed approximately 10% of the amount of claudin-2 protein in control cells, whereas F4, F5, and Tl clones expressed comparable amounts of claudin-2 protein with control cells (Fig. 7A). To confirm the exogenous claudin-2 localization in F1–5 clones, the clones were co-cultured with control cells and analyzed by immunofluorescence microscopy. Claudin-2 staining was barely detectable at TJs in F1–3 clones, whereas claudin-2 staining in F4 and F5 clones was clearly detectable at TJs, although the signal was weaker than in control cells (Fig. 7B).

**Fig 8:**
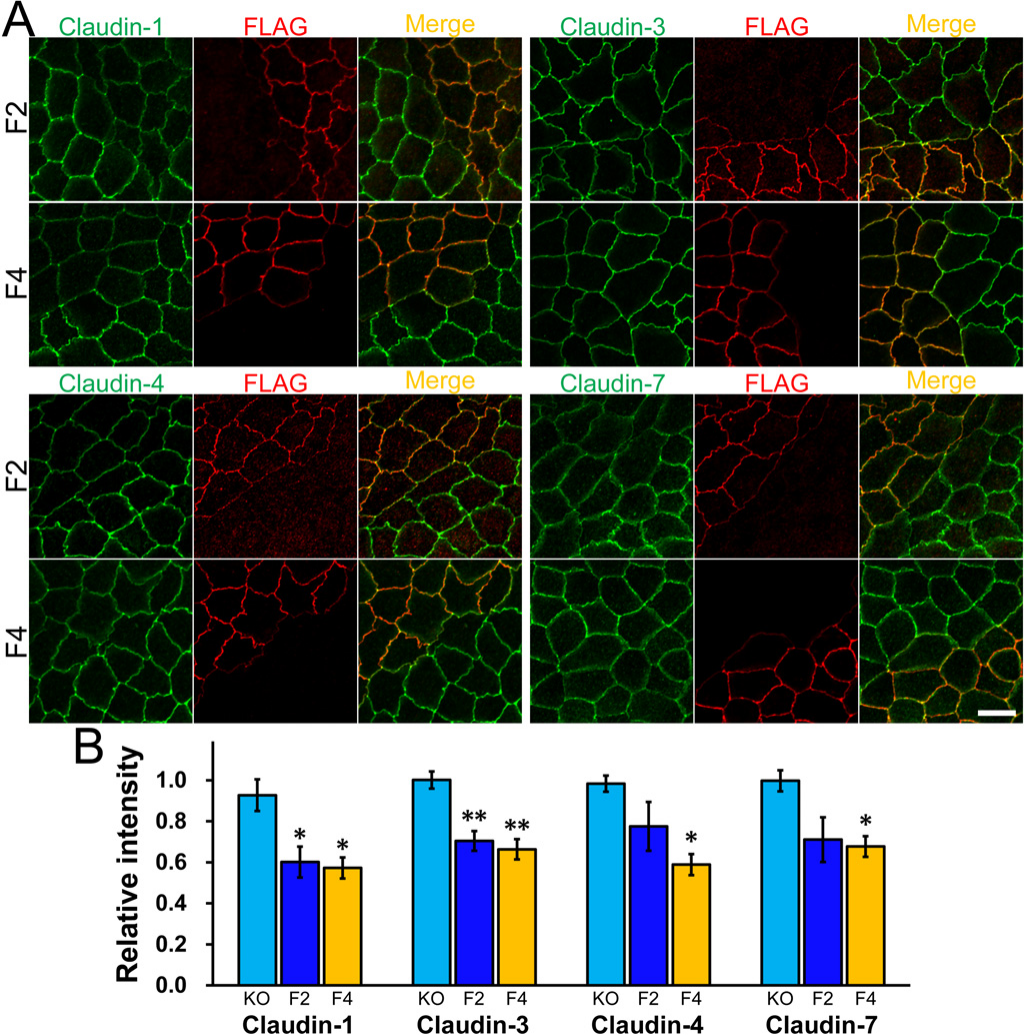
Effects of claudin-2 re-expression on the localization of other claudins in claudin-2 knockout cells. (A) Immunofluorescence analysis of claudins and FLAG in co-culture of claudin-2 knockout cells and F2 or F4 clones. Claudin-1, -3, -4, and -7 staining at TJs in F2 and F4 clones was weaker than those in claudin-2 knockout cells. Scale bar = 10 μm. (B) Quantification analysis of the signal intensity of claudins at TJs in F2 and F4 clones. The signal intensity of claudin-1, -3, -4, and -7 at TJs in F2 and F4 clones was compared with that in claudin-2 knockout cells. N = 4 for each experiment. * *p* < 0.05, ** *p* < 0.01 compared with claudin-2 knockout cells.

The localization of other claudins at TJs in F2 and F4 clones was analyzed by co-culture experiments with claudin-2 knockout cells (Fig. 8) and with control cells (S6 Fig.). Claudin-1, -3, -4, and -7 staining at TJs in F2 and F4 clones was weaker than those in claudin-2 knockout cells, although the signal intensity of claudin-4 and -7 at TJs in F2 clone did not reach a level of significant difference compared with claudin-2 knockout cells (Fig. 8). On the other hand, no significant difference of the signal intensity of claudin-1, -3, -4 and -7 at TJs was detected between F2 and F4 clones and control cells by the quantification method used in this study (S6 Fig.). These results indicate that claudin-2 expression restores the increased localization of other claudins at TJs in claudin-2 knockout cells.

**Fig 9:**
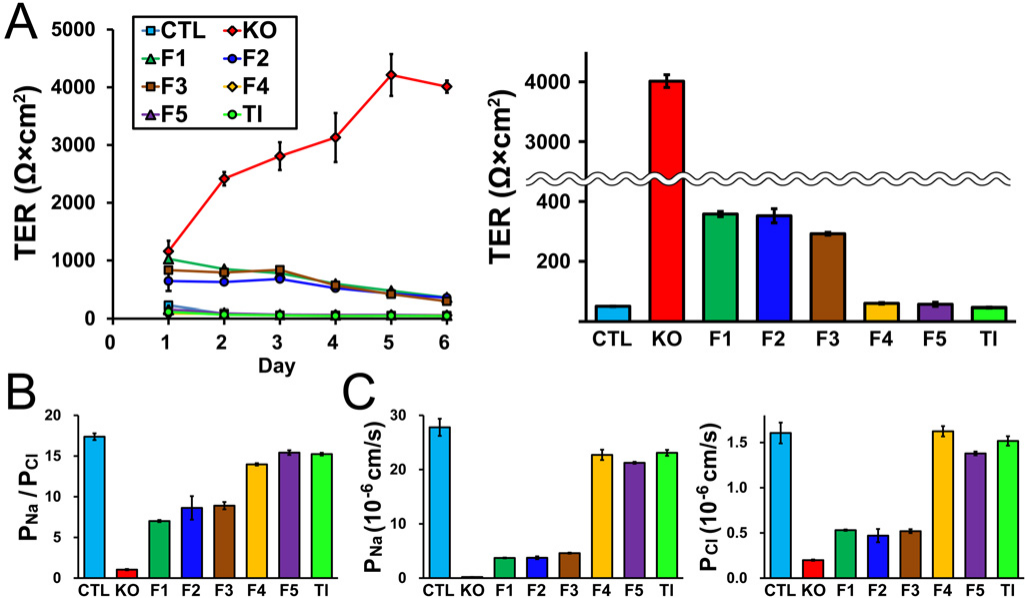
Claudin-2 re-expression restores TJ barrier functions in claudin-2 knockout cells. (A) Time course of TER and TER values at 6 days after seeding on filter inserts in control cells, claudin-2 knockout clone (KO), F1–5 clones and Tl clone. F1–3 clones showed comparable TER to the knockout clone 1 day after seeding on filter inserts, but the TER then decreased with time in contrast to the knockout clone. (B) *P*_Na_/*P*_Cl_ in control cells, claudin-2 knockout clone (KO), F1–5 clones and Tl clone. (C) *P*_Na_ and *P*_Cl_ in control cells, claudin-2 knockout clone (KO), F1–5 clones and Tl clone. N = 3–5 for each experiment.

Next, the electrophysiological properties of these clones were investigated. TER in F4, F5, and Tl clones showed similar values and time course with the TER in control cells (Fig. 9A). In contrast, TER in F1–3 clones 1 day after seeding on filter inserts was comparable to the TER in the claudin-2 knockout clone (F1, 1027 ± 6 Ω·cm^2^; F2, 643 ± 172 Ω·cm^2^; F3, 832 ± 20 Ω·cm^2^ vs KO, 1158 ± 182 Ω·cm^2^), but the TER then decreased with time in contrast to the claudin-2 knockout clone. TER values at 6 days after seeding on filter inserts were less than 10% of the TER value in the claudin-2 knockout clone (F1, 358 ± 9 Ω·cm^2^; F2, 352 ± 24 Ω·cm^2^; F3, 292 ± 5 Ω·cm^2^ vs KO, 4011 ± 106 Ω·cm^2^). F4, F5, and Tl clones had similar *P*_Na_/*P*_Cl_, *P*_Na_, and *P*_Cl_ values with control cells while a partial restoration of *P*_Na_/*P*_Cl_, *P*_Na_, and *P*_Cl_ was observed in F1–3 clones (Fig. 9B and C). These results suggest that claudin-2 re-expression restores TER and charge selectivity of claudin-2 knockout cells dependent upon claudin-2 protein levels, and the effects of a small amount of claudin-2 on TER appear at least 2 days after culture on filter inserts.

### Claudin-2 is less efficiently localized at TJs between control and claudin-2 knockout cells

As shown in Fig. 1, claudin-2 staining was diffuse at TJs between control cells and presumed claudin-2 knockout cells. This result is different from the cases of ZO-1, -2, and -3 (ZO proteins); ZO proteins showed slightly weak but clear staining at TJs between control and their respective knockout cells [19]. We therefore investigated claudin-2 localization at TJs between control and claudin-2 knockout cells in detail.

**Fig 10:**
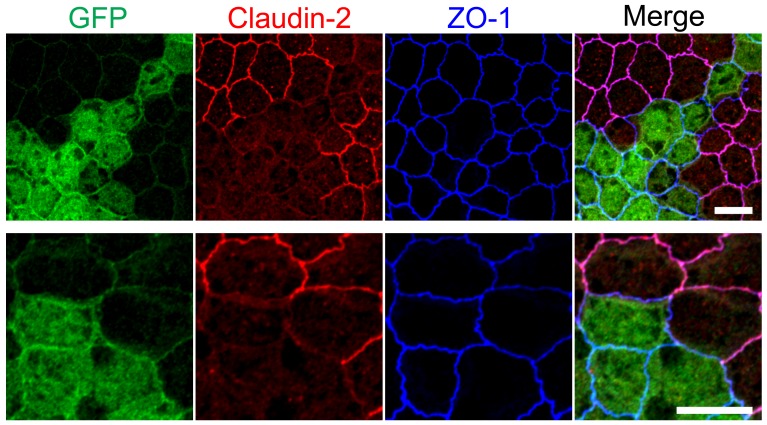
Claudin-2 localization at TJs between control and claudin-2 knockout cells. Immunofluorescence analysis of GFP, claudin-2 and ZO-1 in co-culture of control MDCK II cells and claudin-2 knockout cells expressing EGFP. Claudin-2 staining was diffuse at TJs between control and claudin-2 knockout cells labeled with EGFP. Scale bars = 10 μm.

First, we established a claudin-2 knockout clone stably expressing Enhanced Green Fluorescent Protein (EGFP) to label the knockout cells, and observed claudin-2 localization at TJs between control and knockout cells by co-culture experiments. Predictably, claudin-2 staining was diffuse at TJs between control and claudin-2 knockout cells labeled with EGFP (Fig. 10).

**Fig 11:**
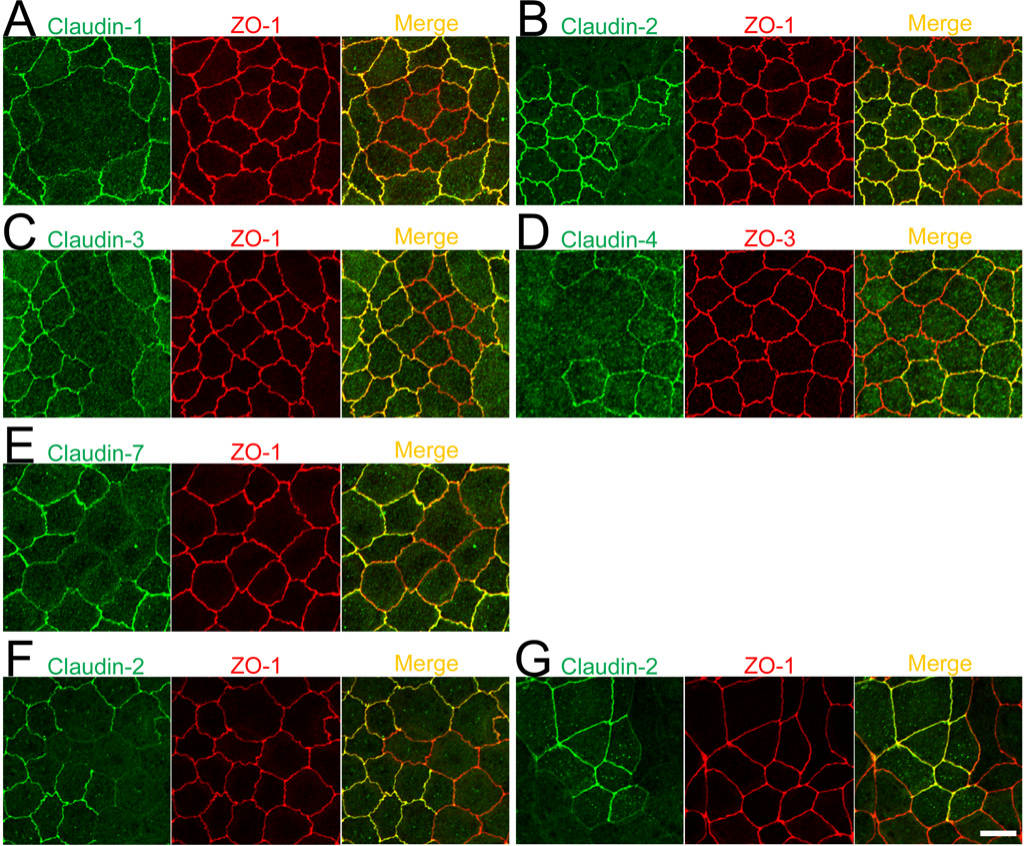
Localization of claudins at TJs between control and their respective knockout cells. (A–E) Immunofluorescence analysis of claudins and ZO-1 or ZO-3 in MDCK II cells transfected with the TALEN constructs for claudin-1, -2, -3, -4, or-7 gene knockouts. After transfection, cells were subcultured on filter inserts for 4 days before the analysis of claudin-2 and -3 (B and C), and for 2 days before the analysis of claudin-1, -4, and -7 (A, D, and E). (F) Immunofluorescence analysis of claudin-2 and ZO-1. TALEN constructs for the claudin-2 gene knockout were transfected into MDCK II cells, which were subcultured on filter inserts for 2 days before analysis. (G) Immunofluorescence analysis of claudin-2 and ZO-1 in a co-culture of wild-type and claudin-2 expressing MDCK I cells. Cells were cultured on filter inserts for 4 days before analysis. Scale bar = 10 μm.

To confirm whether the diffuse localization at TJs between control and knockout cells is specific for claudin-2, we constructed TALENs to knockout claudin-1, -3, -4, and -7 (S7 Fig.), and conducted knockouts of these claudins. As shown in Figs. 3 and 4, the staining of some claudins at TJs was not clear in wild-type MDCK II cells; however, the staining of claudin-1, -4, and -7 was clearer at TJs during the early days of culture on filter inserts whereas claudin-2 staining at TJs became more obvious over time (S8 Fig.). Therefore, immunofluorescence analysis of claudin-1, -4, and -7 was performed on cell monolayers cultured for 2 days on filter inserts. In contrast to claudin-2, clear staining of claudin-1, -3, and -4 was observed at TJs between control and their respective knockout cells, while claudin-7 staining was slightly diffuse at TJs between control and claudin-7 knockout cells (Fig. 11A–E). Claudin-2 localization in cell monolayers cultured for 2 days on filter inserts was also diffuse at TJs between control and claudin-2 knockout cells (Fig. 11F). To confirm whether the diffuse localization of claudin-2 at TJs between control and claudin-2 knockout cells is specific for MDCK II cells, we established claudin-2 expressing MDCK I cells that lack the endogenous expression of claudin-2 and investigated claudin-2 localization in co-culture experiments with wild-type MDCK I cells. Claudin-2 localization was also diffuse at TJs between wild-type and claudin-2 expressing MDCK I cells (Fig. 11G).

**Fig 12:**
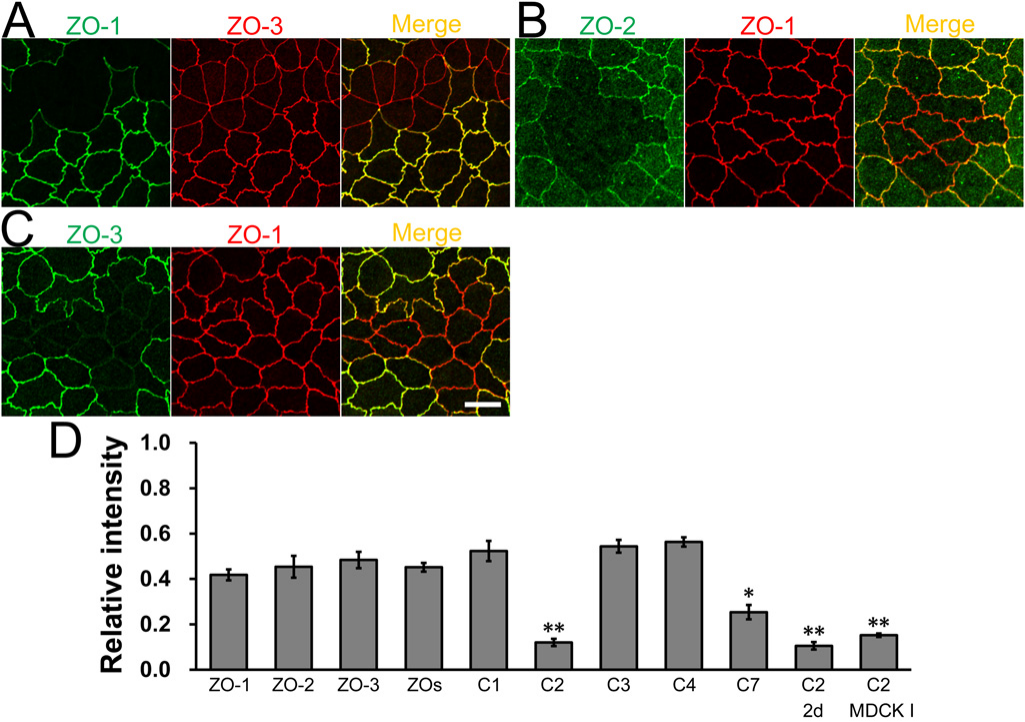
Quantification analysis of the signal intensity of claudins and ZO proteins at TJs between control and their respective knockout cells. (A–C) Immunofluorescence analysis of ZO proteins in MDCK II cells transfected with TALEN constructs for ZO-1, -2, or-3 gene knockouts. After transfection, cells were subcultured on filter inserts for 4 days before analysis. ZO-1 knockout cells showed a straight shape of cell-cell contacts consistent with a previous study [19]. Scale bar = 10 μm. (D) Quantification analysis of the signal intensity of claudins and ZO proteins at TJs between control and their respective knockout cells. The signal intensity of these proteins between control cells and that between control and their respective knockout cells was measured, and the relative signal intensity of each protein was calculated as described in *Materials and Methods*. The relative intensity of claudins was compared with the average of the relative intensity of ZO proteins (ZOs). N = 3–6 for each experiment. * *p* < 0.05, ** *p* < 0.01 compared with ZOs.

To compare quantitatively the localization of claudins at TJs between control and their respective knockout cells, we measured the signal intensity of claudins at TJs as described above, and calculated the ratio of signal intensity between control cells to that between control and knockout cells (relative intensity; see *Materials and Methods* for details). To compare the relative intensity, we first quantified the relative intensity of ZO-1, -2, and -3 using TALENs constructed in a previous study [19] (Fig. 12). The signal intensity of ZO proteins at TJs between control and knockout cells was approximately 50% of that between control cells, and the average of the relative intensity of ZO proteins was 0.45 ± 0.03 (ZOs in Fig. 12D). The relative intensity of claudin-1, -3, and -4 was similar to that of ZO proteins. In contrast, the relative intensity of claudin-2 and -7 was significantly lower compared with ZO proteins. These results suggest that the efficiency of claudin localization at TJs between control and knockout cells varies among claudins, and claudin-2 and -7 are less efficiently localized at TJs between control and their respective knockout cells.

## Discussion

Claudin-2 has been reported to form high conductive cation pores in TJs and be involved in paracellular water permeability [10–12,30]. In this study, we succeeded in establishing claudin-2 knockout clones in MDCK II cells using the TALEN technique. To improve the efficiency of knockout clone selection, we transiently administered G418 and puromycin as described previously [19], and established five independent claudin-2 knockout clones.

Immunoblot analysis with an anti-claudin-2 antibody demonstrated the appearance of faint bands with a molecular mass lower than that of wild-type claudin-2 in claudin-2 knockout clones. Because the open reading frame of canine claudin-2 gene has an in-frame ATG sequence at the 24th codon, these faint bands might reflect an artificial peptide formed by translation using the 24th ATG codon of the claudin-2 gene as its initiating codon, which lacks the intracellular N terminus and most of the first transmembrane helix of claudin-2. Predicted molecular masses of wild-type claudin-2 and the artificial peptide are 24.5 kDa and 22.1 kDa, respectively, and the wild-type claudin-2 band and faint bands correspond to these predicted molecular masses. It is difficult to clarify the nature of the faint bands, but the peptide represented by the faint bands is not likely to have significant effects on the localization of claudins or the electrophysiological properties of MDCK II cells, because claudin-2 knockout clones showed prominent changes in these phenotypes and claudin-2 re-expression restored these changes.

Genomic PCR analysis indicated that TALEN DNAs were not integrated into the chromosome in claudin-2 knockout clones as seen in ZO-1 knockout clones we established previously [19]. Because the persistent expression of TALENs has potential adverse effects including increased frequency of off-target cleavage [31], it is ideal to establish knockout clones in which TALENs are not integrated into the chromosome. This is likely to be more important for double- and triple-gene knockouts by TALENs, because persistent TALEN expression in cells can form *Fok*I dimers at off-target sites with TALENs transfected for the knockout of another gene. Therefore, the knockout clones established in this and previous studies are likely to be useful for double- and triple-knockout analyses.

Claudin-2 forms high conductive cation pores in TJs, and the suppression of claudin-2 expression by RNA interference in MDCK II cells induced a three-fold increase in TER [13,14]. However, in contrast, claudin-2 knockout increased TER by more than 50-fold in MDCK II cells, comparable to that in high-resistance strains of MDCK cells [32]. Because the rescue experiments indicated that a small amount of claudin-2 markedly decreased TER in claudin-2 knockout MDCK II cells, the remaining claudin-2 expression in knockdown analysis was likely to cause the difference of TER in claudin-2 knockdown and knockout cells. This is similar to that seen for ZO-1 knockout, where noticeable changes in ZO-1 knockout MDCK cells unseen in previous ZO-1 knockdown studies (changes in myosin organization at cell-cell contacts and localization of TJ proteins) were restored by small amounts of ZO-1 [19]. The results of the current and previous studies suggest the importance of knockout analysis in cultured cells.

In tight epithelia such as claudin-2 knockout MDCK II cells, it is necessary to be careful when interpreting electrophysiological measurements. Transepithelial movements of substances occur via two routes: transcellular and paracellular pathways. TER and dilution potentials (reflection of charge selectivity) are affected by these two pathways, and the contribution of the transcellular pathway to TER and dilution potentials in tight epithelia is relatively larger and cannot be ignored compared with those in leaky epithelia [33,34]. Therefore, the effect of claudin-2 knockout on the electrical resistance of the paracellular pathway in MDCK II cells is thought to be still larger than the measured difference of TER between control and claudin-2 knockout cells. In addition, although claudin-2 knockout decreased cation selectivity in MDCK II cells consistent with previous studies [10–13], it is necessary to take into consideration the contribution of transcellular pathway in the measured values of charge selectivity (*P*_Na_/*P*_Cl_), *P*_Na_, and *P*_Cl_ in claudin-2 knockout MDCK II cells.

Interestingly, the TER in clones expressing a small amount of claudin-2 1 day after seeding on filter inserts was similar to TER in the claudin-2 knockout clone and the TER then gradually decreased with time, suggesting that claudin-2 pore formation in TJs occurs later than the development of TER by TJ strand formation with other claudins in these clones. The time lag of claudin-2 pore formation in TJs might also occur in wild-type MDCK II cells, because the TER of wild-type MDCK II cells also decreases with time after culture on filter inserts [29], and claudin-2 localization at TJs became clearer over time during culture on filter inserts whereas the localization of claudin-1, -4, and -7 was clearer during the early days of culture. Further analysis using a combination of electrophysiological, morphological, and molecular biological approaches is required to understand the temporal differences of claudins during TJ strand formation.

The localization of claudin-1, -3, -4, and -7 at TJs in MDCK II cells was increased in claudin-2 knockout cells, suggesting these claudins were additionally incorporated into TJ strands in the absence of claudin-2. Because claudin-2 knockout cells had a very high TER, claudin-1, -3, -4, and -7 are not likely to form pores with high ion conductivity in TJs and these claudins also contribute to the high TER in claudin-2 knockout cells. Of note, claudin-7 localization increased at TJs in claudin-2 knockout cells. The localization of some claudins is not restricted to TJs but rather is distributed along the basolateral membrane [35], and claudin-7 is known to be mostly located along the basolateral membrane in MDCK cells [14] and in tissues [36–38]. In contrast, claudin-7 was clearly detected at TJs in claudin-2 knockout cells. Few studies have shown an increase in the localization of claudins at TJs by manipulation, including treatment with ouabain (Na^+^/K^+^-ATPase inhibitor) [39], osmotic changes [40], and epithelial cell adhesion molecule (EpCAM) knockdown [41], and the current study suggests that claudin-2 itself also affects the localization of other claudins at TJs including claudin-7. Further analysis of claudin localization in claudin knockout cells, other than claudin-2, is required to aid our understanding of this phenomenon.

Claudin-2 was localized at TJs less efficiently when the adjacent cells did not express claudin-2. Claudins are thought to polymerize into strands that bind strands in adjacent cells at TJs (*trans*-binding) [4,42]. Thus, the low efficiency of claudin-2 localization at TJs between control and claudin-2 knockout cells indicates the possibility that the *trans*-binding ability of claudin-2 to strands not containing claudin-2 is weaker than to strands with claudin-2. Although homotypic and heterotypic *trans*-binding ability between several pairs of single claudins has been investigated using various cells lacking endogenous TJs [24,43–45], analysis of the *trans*-binding ability of claudins in endogenous TJ strands comprising multiple claudins has not been previously reported. Because the first and second extracellular domains contribute to homotypic and heterotypic *trans*-binding of claudins [44,46,47], further analysis of the responsible domains of claudin-2 that affect claudin-2 localization efficiency at TJs between control and claudin-2 knockout cells might increase our understanding of the *trans*-binding ability of claudins in endogenous TJ strands.

In conclusion, we established claudin-2 knockout clones in MDCK II cells and revealed that claudin-2 independently determines the ‘leaky’ property of TJs in MDCK II cells. Our results suggest the effectiveness and necessity of knockout analysis in cultured cells for future studies.

## Supporting Information

S1 Fig Quantification of TJ protein localization at TJs. Images were opened in Image J 1.43u, and immunofluorescence signals of TJ marker proteins (ZO-1, ZO-3, or occludin) were traced with 0.5-μm-wide freehand lines to build the region of interest.
(TIF)

S2 Fig Immunoblots of claudin-2 in control MDCK II cells and claudin-2 knockout clones. (A) Immunoblots of claudin-2 in control MDCK II cells and claudin-2 knockout clones with rabbit anti-claudin-2 pAb. (B) Immunoblots of claudin-2 in control MDCK II cells and claudin-2 knockout clones with mouse anti-claudin-2 mAb.
(TIF)

S3 Fig Chromatograms of sequences around the TALEN targeting site in control cells and claudin-2 knockout clones. (A) Chromatograms of sequences around the TALEN targeting site in control cells and claudin-2 knockout clones. PCR products of the TALEN targeting site from control cells and claudin-2 knockout clones were directly subjected to DNA sequencing analysis (control, CTL; knockout clones, KO 1–5). Chromatograms of the sequences for KO 2–5 clones showed mixed peak arrays, thus PCR products from KO 2–5 clones were cloned into a plasmid vector and subjected to sequence analysis. (B) DNA sequences of the TALEN targeting site in allele 1 of the KO 5 clone. A deletion of 52 base pairs was observed in the allele.
(TIF)

S4 Fig Effect of claudin-2 knockout on the localization of other claudins. Immunofluorescence analysis of claudins in co-culture of control MDCK II cells and claudin-2 knockout clone 2 (KO 2). Scale bar = 10 μm.
(TIF)

S5 Fig Localization of claudins in z-axis plane in control and claudin-2 knockout cells. Immunofluorescence analysis of claudins and occludin in co-culture of control MDCK II cells and claudin-2 knockout clone 2 (KO 2) in z-axis plane. Scale bar = 5 μm.
(TIF)

S6 Fig Claudin-2 re-expression restores the localization of other claudins in claudin-2 knockout cells. (A) Immunofluorescence analysis of claudins and FLAG in co-culture of control MDCK II cells and F2 or F4 clones. Signals of claudin-1, -3, -4, and -7 at TJs in F2 and F4 clones were similar to those in control cells. Scale bar = 10 μm. (B) Quantification analysis of the signal intensity of claudins at TJs in F2 and F4 clones. The signal intensity of claudin-1, -3, -4, and -7 at TJs in F2 and F4 clones was compared with that in control cells, and no significant difference of the signal intensity of these claudins was detected between F2 and F4 clones and control cells. N = 4–5 for each experiment.
(TIF)

S7 Fig Construction of TALENs for the knockout of canine claudin-1, -3, -4, and -7. TALEN binding sites in the claudin-1, -3, -4, and -7 genes. TALENs were designed to target the initiating codon or the immediate following regions. The left and right arms of TALEN targeting sites are indicated in blue and the spacer regions are indicated in red. The initiating codons are shaded.
(TIF)

S8 Fig Immunofluorescence analysis of claudins in wild-type MDCK II cells cultured for 2 and 4 days on filter inserts. Immunofluorescence analysis of claudin-1, -2, -3, -4, and -7 in wild-type MDCK II cells cultured for 2 and 4 days on filter inserts. Scale bar = 10 μm.
(TIF)
