# Supplementary figures and images for: Claudin-2 Knockout by TALEN-Mediated Gene Targeting in MDCK Cells: Claudin-2 Independently Determines the Leaky Property of Tight Junctions in MDCK Cells
Source: PLoS One. 2015 Mar 17;10(3):e0119869. doi: 10.1371/journal.pone.0119869 (PMC4363821; doi:10.1371/journal.pone.0119869)

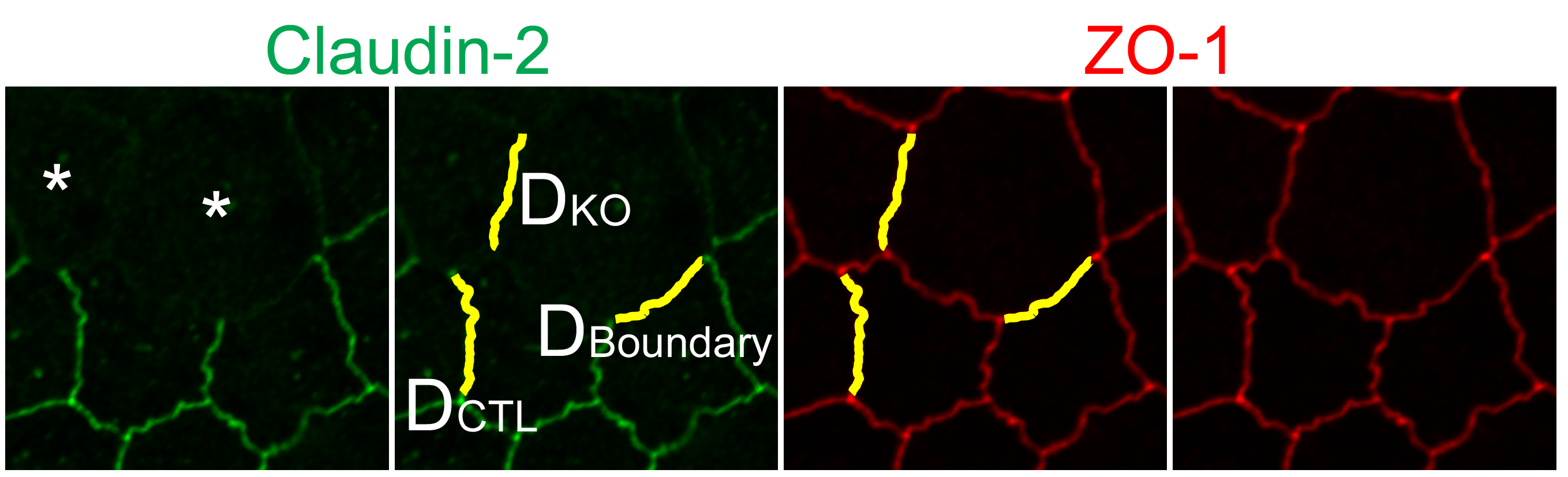

Supplement: S1 Fig — Images were opened in Image J 1.43u, and immunofluorescence signals of TJ marker proteins (ZO-1, ZO-3, or occludin) were traced with 0.5-μm-wide freehand lines to build the region of interest. (TIF) [file pone.0119869.s001.tif]

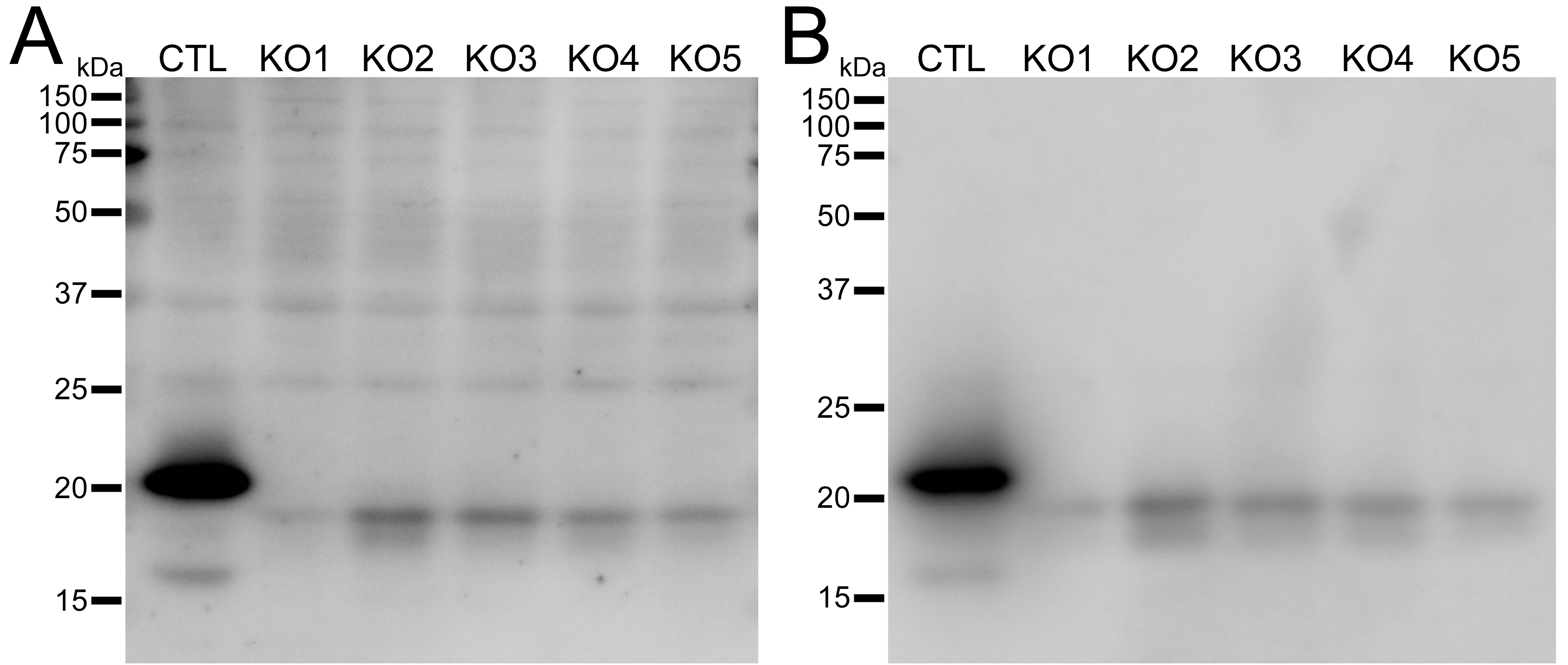

Supplement: S2 Fig — (A) Immunoblots of claudin-2 in control MDCK II cells and claudin-2 knockout clones with rabbit anti-claudin-2 pAb. (B) Immunoblots of claudin-2 in control MDCK II cells and claudin-2 knockout clones with mouse anti-claudin-2 mAb. (TIF) [file pone.0119869.s002.tif]

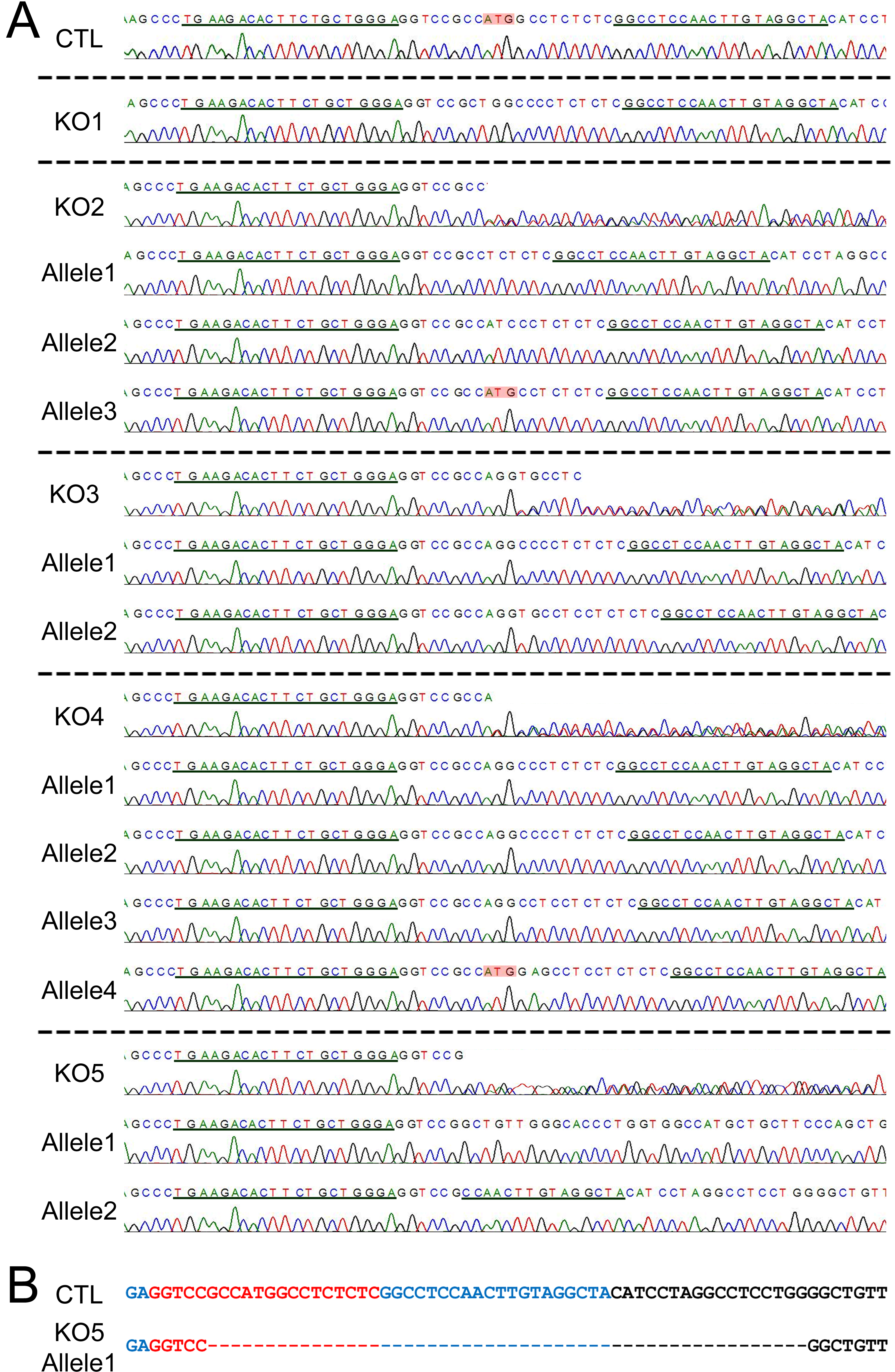

Supplement: S3 Fig — (A) Chromatograms of sequences around the TALEN targeting site in control cells and claudin-2 knockout clones. PCR products of the TALEN targeting site from control cells and claudin-2 knockout clones were directly subjected to DNA sequencing analysis (control, CTL; knockout clones, KO 1–5). Chromatograms of the sequences for KO 2–5 clones showed mixed peak arrays, thus PCR products from KO 2–5 clones were cloned into a plasmid vector and subjected to sequence analysis. (B) DNA sequences of the TALEN targeting site in allele 1 of the KO 5 clone. A deletion of 52 base pairs was observed in the allele. (TIF) [file pone.0119869.s003.tif]

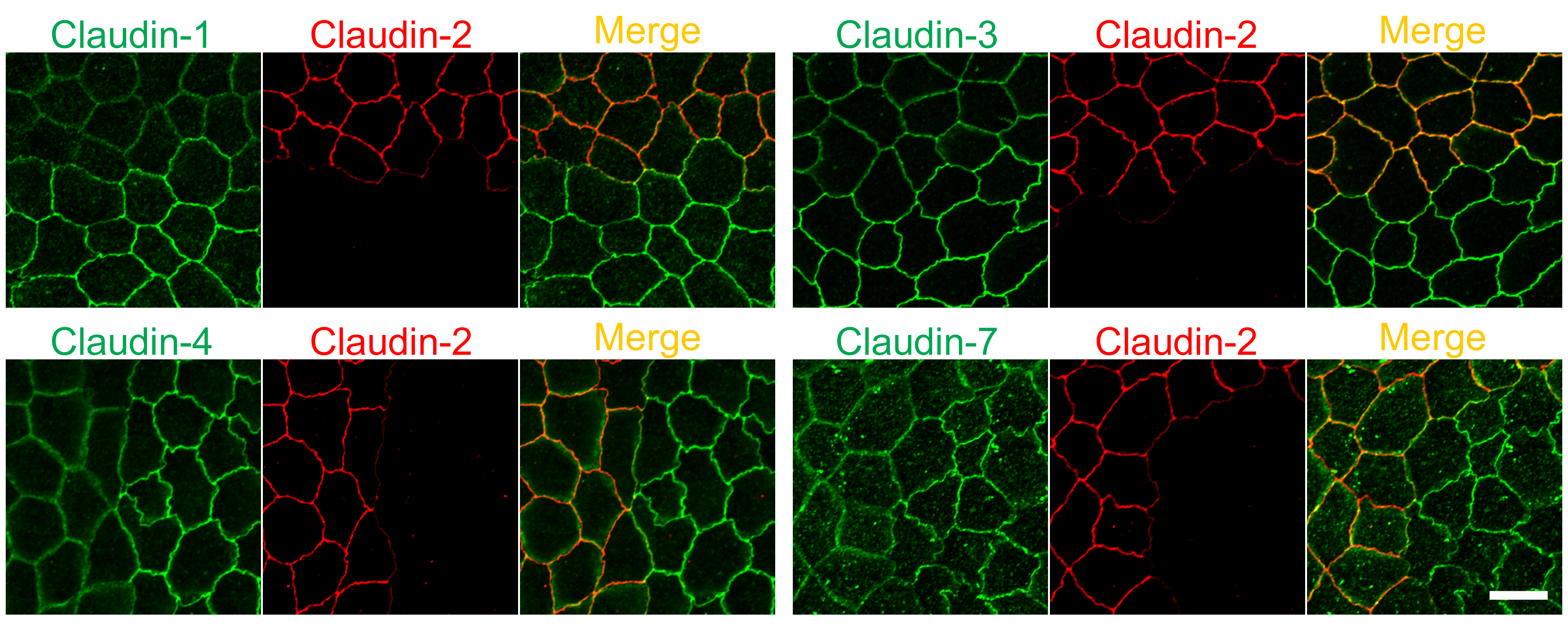

Supplement: S4 Fig — Immunofluorescence analysis of claudins in co-culture of control MDCK II cells and claudin-2 knockout clone 2 (KO 2). Scale bar = 10 μm. (TIF) [file pone.0119869.s004.tif]

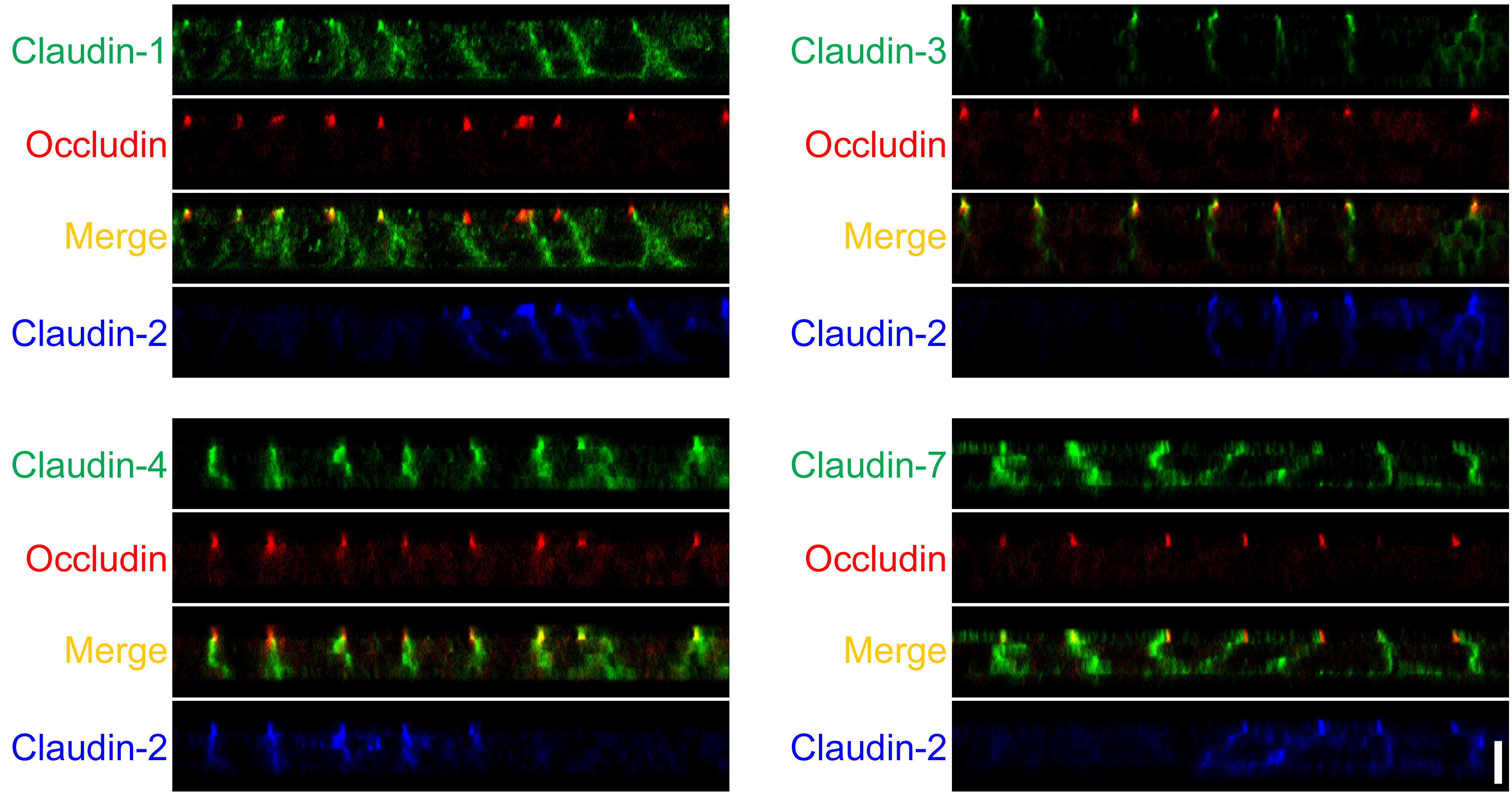

Supplement: S5 Fig — Immunofluorescence analysis of claudins and occludin in co-culture of control MDCK II cells and claudin-2 knockout clone 2 (KO 2) in z-axis plane. Scale bar = 5 μm. (TIF) [file pone.0119869.s005.tif]

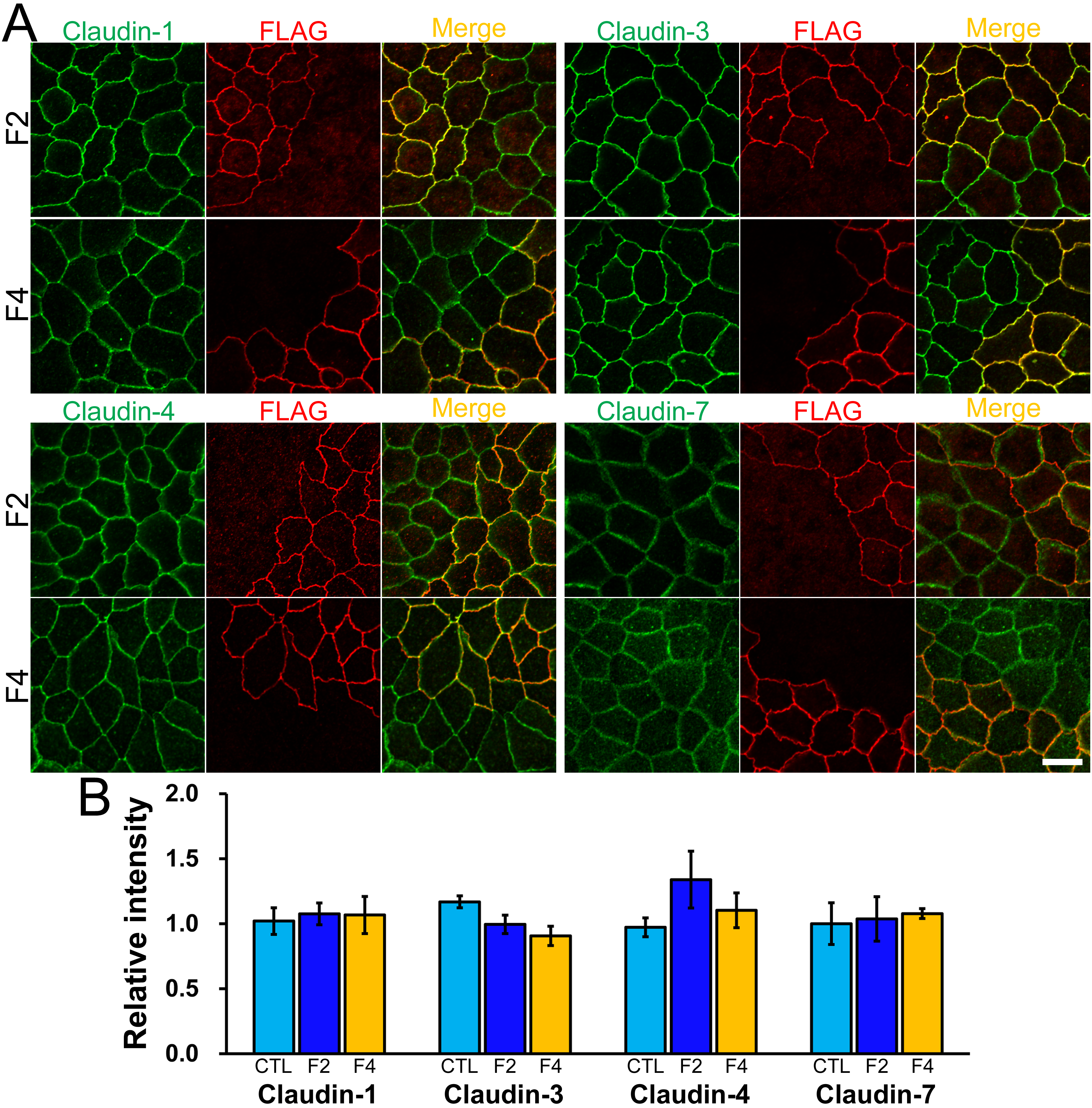

Supplement: S6 Fig — (A) Immunofluorescence analysis of claudins and FLAG in co-culture of control MDCK II cells and F2 or F4 clones. Signals of claudin-1, -3, -4, and -7 at TJs in F2 and F4 clones were similar to those in control cells. Scale bar = 10 μm. (B) Quantification analysis of the signal intensity of claudins at TJs in F2 and F4 clones. The signal intensity of claudin-1, -3, -4, and -7 at TJs in F2 and F4 clones was compared with that in control cells, and no significant difference of the signal intensity of these claudins was detected between F2 and F4 clones and control cells. N = 4–5 for each experiment. (TIF) [file pone.0119869.s006.tif]

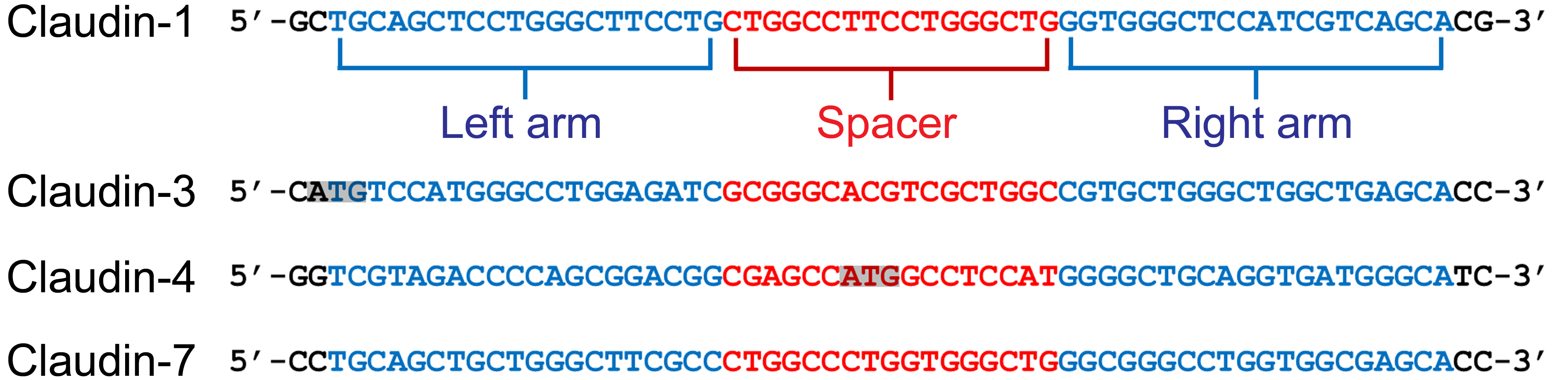

Supplement: S7 Fig — TALEN binding sites in the claudin-1, -3, -4, and -7 genes. TALENs were designed to target the initiating codon or the immediate following regions. The left and right arms of TALEN targeting sites are indicated in blue and the spacer regions are indicated in red. The initiating codons are shaded. (TIF) [file pone.0119869.s007.tif]

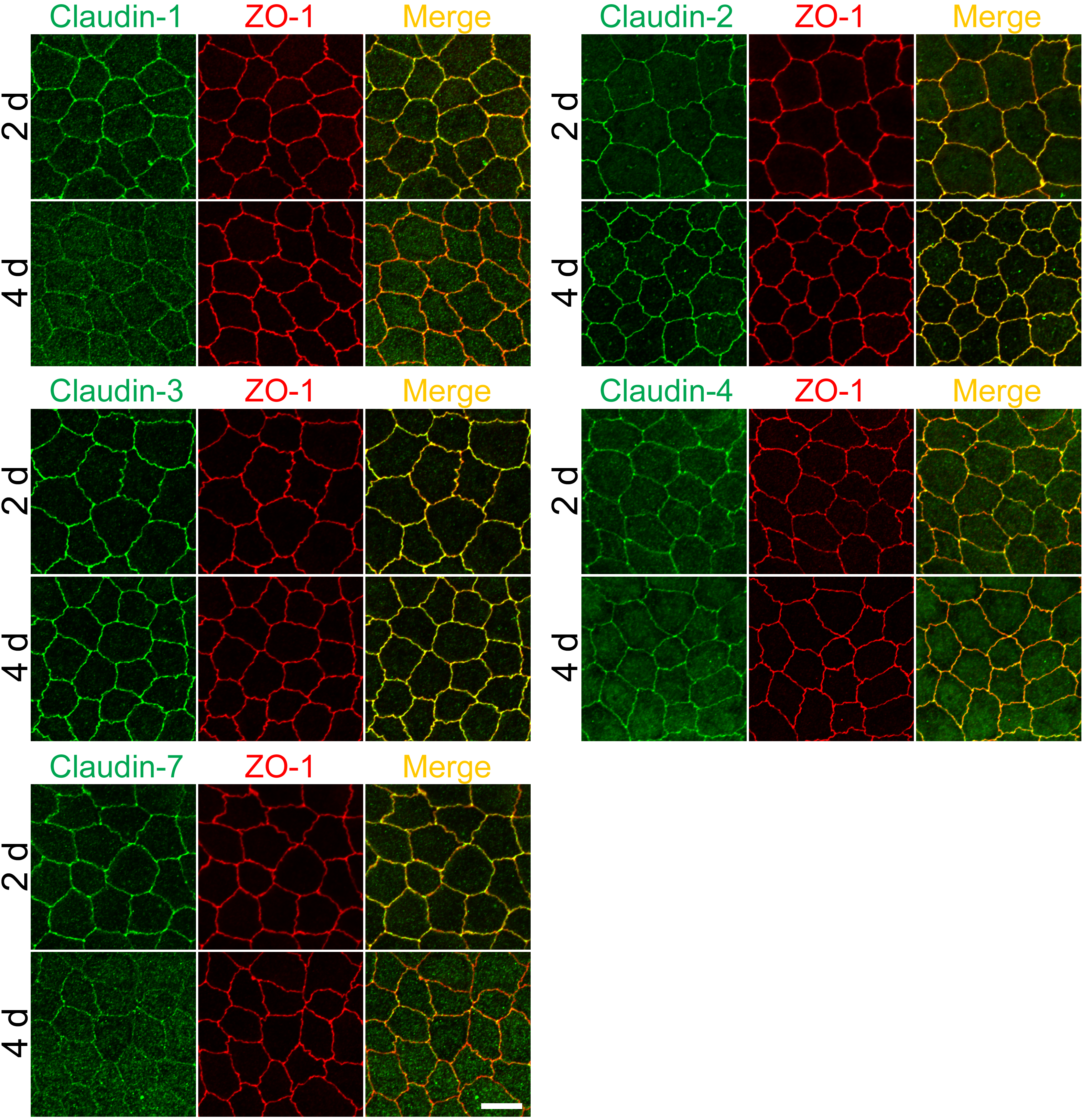

Supplement: S8 Fig — Immunofluorescence analysis of claudin-1, -2, -3, -4, and -7 in wild-type MDCK II cells cultured for 2 and 4 days on filter inserts. Scale bar = 10 μm. (TIF) [file pone.0119869.s008.tif]
